# New Concept of Power Generation Using TEGs: Thermal Modeling, Parametric Analysis, and Case Study

**DOI:** 10.3390/e22050503

**Published:** 2020-04-27

**Authors:** Ahmad Faraj, Hassan Jaber, Khaled Chahine, Jalal Faraj, Mohamad Ramadan, Hicham El Hage, Mahmoud Khaled

**Affiliations:** 1 Energy and Thermofluid Group, Lebanese International University (LIU), Bekaa 1801, Lebanon; ahmad.faraj@liu.edu.lb; 2 Energy and Thermofluid Group, International University of Beirut BIU, Beirut 1001, Lebanon; hassan.jaber@liu.edu.lb (H.J.); jalal.faraj@liu.edu.lb (J.F.); mohamad.ramadan@liu.edu.lb (M.R.); hisham.elhage@liu.edu.lb (H.E.H.); 3 Electrical and Computer Engineering Department, Beirut Arab University, Debbieh 115020, Lebanon; k.shahine@bau.edu.lb; 4 Associate Member at FCLAB, CNRS, University of Bourgogne Franche-Comté, 90018 Belfort CEDEX, France; 5 Interdisciplinary Energy Research Institute (PIERI), University Paris Diderot, Sorbonne Paris Cite, 75000 Paris, France

**Keywords:** new concept, power generation, TEG, HVAC, sun irradiation

## Abstract

In this manuscript, an innovative concept of producing power from a thermoelectric generator (TEG) is evaluated. This concept takes advantage of using the exhaust airflow of all-air heating, ventilating, and air-conditioning (HVAC) systems, and sun irradiation. For the first step, a parametric analysis of power generation from TEGs for different practical configurations is performed. Based on the results of the parametric analysis, recommendations associated with practical applications are presented. Therefore, a one-dimensional steady-state solution for the heat diffusion equation is considered with various boundary conditions (representing applied configurations). It is revealed that the most promising configuration corresponds to the TEG module exposed to a hot fluid at one face and a cold fluid at the other face. Then, based on the parametric analysis, the innovative concept is recognized and analyzed using appropriate thermal modeling. It is shown that for solar radiation of 2000 W/m^2^ and a space cooling load of 20 kW, a 40 × 40 cm^2^ flat plate is capable of generating 3.8 W of electrical power. Finally, an economic study shows that this system saves about $6 monthly with a 3-year payback period at 2000 W/m^2^ solar radiation. Environmentally, the system is also capable of reducing about 1 ton of CO_2_ emissions yearly.

## 1. Introduction

Heat recovery from waste energy is an essential method that is generally used for energy management [1,2,3,4,5]. Nowadays, the conversion of waste heat into electrical power is one of the best approaches. It consists of using thermoelectric power generators (TEGs). A TEG is a solid component that provides direct energy due to the variation of the surrounding thermal behavior, and electrical power can then be generated based on the “Seebeck effect” and temperature gradient [6,7,8]. Thermoelectric technology had become popular in heat recovery applications and it was implemented with various sources such as internal combustion engines [9,10,11,12], stoves [13,14,15], vehicles [16,17,18,19], biomass gasifiers [20], chimneys [21,22], diesel engines [23,24,25,26], and fuel cells [27]. 

Jaber et al. [21] performed a theoretical study on a hybrid heat recovery system that utilizes exhaust gases of a domestic chimney to produce hot water and generate electricity using TEGs. Optimization analysis on the location of TEGs within the system was performed. Six cases were considered by which TEGs were located on the inner or outer surface of the pipe or tank or on all surfaces. Results showed that as TEGs were placed nearer to the exhaust gases, the generated power was increased and the water temperature was decreased. As TEGs located on the inner surface of the pipe (in direct contact with exhaust gases) water temperature achieved 76 ℃ and 35 W electric power was generated. Moreover, economic and environmental studies were performed showing that the most cost-effective configuration was achieved when TEGs were placed in direct contact with exhaust gases. However, changing the location of TEGs did not highly affect the reduction of CO_2_ gas. Orr et al. [28] also reviewed automotive waste heat recovery using TEGs and heat pipes.

Typically, the heat source is obtained from the exhaust gases by which TEGs are attached at the surface of the exhaust pipe; while cooling water is utilized as a heat sink. It should be noted that BMW, Ford, Renault, and Honda have expressed interest in TEG technology. BMW achieved 750 W electric power from 20 W rated TEGs; Whereas Ford reached 400 W from 4.6 kg thermoelectric materials. About 1 kW is predicted to be generated from Renault diesel truck. Honda generated 500 W from 32 TEG of 30 mm × 30 mm dimensions which led to a 3% reduction in fuel consumption [28].

Rahman et al. [29] employed an effective approach to recover heat from exhaust gases of an internal combustion engine using TEGs. The waste recovery system supplies hot air (between 70 and 80 °C) to the engine. The recovery system is composed of fuzzy intelligent controlled micro-faucet emission gas recirculation (MiF-EGR) and TEGs. It was shown that TEGs were could reduce the load of the alternator by 10%. In addition, the specific fuel consumption was enhanced by 3% and the brake power increased by 7% which is resulted from heating air [29].

An experimental study coupled with thermal modeling in a heat recovery system that utilizes the exhaust gases of a wood stove to generate electricity was performed by Champier et al. [13]. It was shown that the performance of the TEG is directly dependent on the heat transfer rate at the TEG and the contact thermal resistance generated about 9.5 W electric power. 

Gao et al. [27] presented numerical modeling on heat recovery from a high-temperature polymer electrolyte membrane fuel cell. TEGs were placed inside the walls of a compact plate-fin heat exchanger. A finite element approach was utilized by which each segment of the fluid properties, TEG performance, and heat transfer rate were calculated. 

Ma et al. [20] performed an experimental study on the power generation heat recovery system from a biomass gasifier. Bi_2_Te_3_ thermoelectric generators were utilized to handle the temperature of exhaust gases from the gasifier (473–633 K). The maximum power density reached by the system was 193 W/m^2^ and the maximum power produced by TEGs was 6.1 W. Finally, the energy conversion efficiency ranged between 10.9% and 2.8% at 505 K and 75 K temperature difference respectively.

It is known that heat recovery and renewable energy are the main solutions for the energy crisis. Providing a new system that can utilize both heat recovery and renewable energy is a real challenge. Furthermore, exploring the existing literature of TEG applications reveals that challenges are encountered in applications (actual cases) where significant temperature differences can be generated across thermoelectric generators rather than increasing the efficiency of the thermoelectric generators themselves. In this context, the present study suggests a new concept of power generation from TEGs using the exhaust airflow of all-air heating, ventilating, and air-conditioning (HVAC) systems and sun irradiation. Indeed, heating, ventilating, and air-conditioning (HVAC) systems are essential mechanical systems to ensure thermal comfort [30]. The considerable amounts of air exhausted from all-air HVAC systems can be employed as a heat sink at one side of TEG modules. 

The proposed design uses a cubic oil tank with a flat plate composed of several TEG modules in series at its bottom, where its upper surface is considered semi-transparent and exposed to sun irradiation. Moreover, the bottom plate of the tank is cooled at its lower surface by the exhaust air of the HVAC system while the upper surface of each TEG module is heated by oil, and the oil itself is heated by the transmitted solar irradiation. Consequently, a temperature gradient will be induced and electrical power could be generated. To continue, appropriate thermal modeling of the design is developed and the performance is investigated as a function of the solar radiation available and the cooling load of the conditioned space.

## 2. Theoretical Background

### 2.1. Thermoelectric Power Generation 

**Figure 1:**
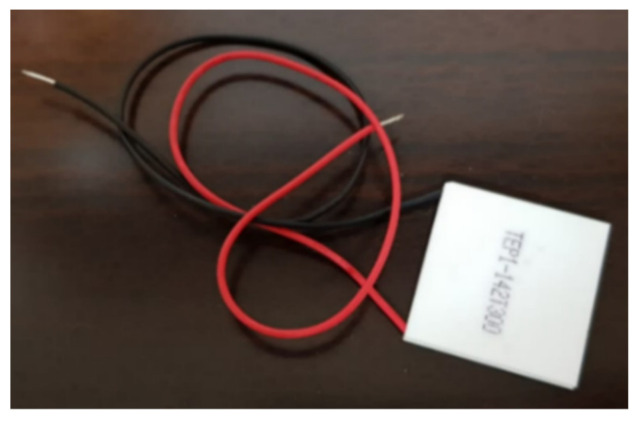
Thermoelectric generator.

Thermoelectric technology is defined as the direct conversion of thermal energy to electric energy [31,32]. Thermoelectric generators (Figure 1) are passive devices that generate electricity when subjected to a temperature gradient.

**Figure 2:**
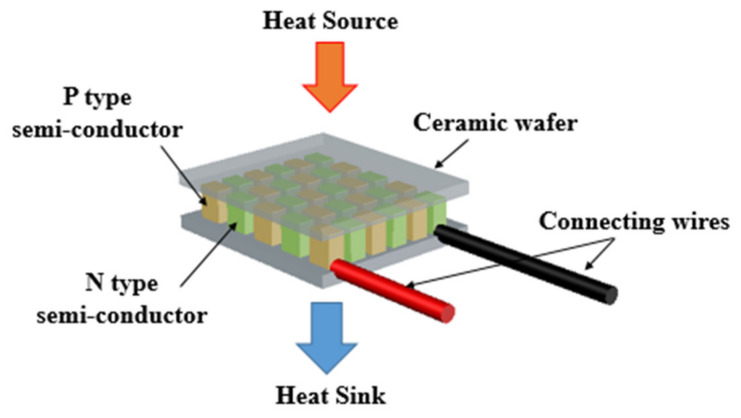
Schematic of the thermoelectric generator (TEG).

It is composed of types P and N semiconductors that are connected electrically in series as shown in Figure 2. The main advantage of such devices is that they do not have any moving parts and are reliable and silent. However, such devices have low efficiency (about 5%) [31]. Based on the Seebeck effect, when a thermoelectric generator is sandwiched between a heat source and heat sink, an induced voltage is generated [32]. The performance of the thermoelectric material is described by the “figure of merit” ZT. The figure of merit depends on the Seebeck coefficient S, electrical conductivity σ, and thermal conductivity k [28].

### 2.2. Heating, Ventilating, and Air Conditioning

**Figure 3:**
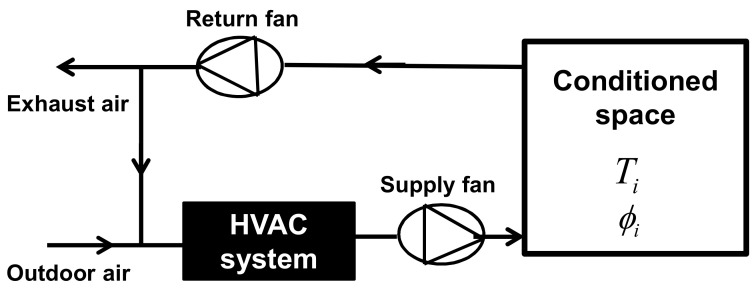
Schematic of the operational mode of all-air heating, ventilating, and air-conditioning (HVAC) system.

There are three main categories of HVAC systems: all-water, water–air, and all-air systems. An all-air system is referred to as such because the temperature and humidity are controlled only by supplying air to the conditioned space. Figure 3 shows a schematic of the operational mode of this category of HVAC system [30].

The assembly of components shown in Figure 3, which is known as an “air handling unit”, has the capability to remove energy from or add energy to airstreams prior to supplying the conditioned spaces with fresh air [30]. It can heat, cool, humidify, dehumidify, clean, and distribute air to the various conditioned spaces in a zone. In addition, outdoor air can be admitted and room air can be exhausted through the air handling system. In general, in for a compromise between comfort and economy, a fraction of the return air (sometimes all the amount of the supplied air is exhausted) from the conditioned spaces is exhausted, and, instead of it, an amount of outdoor air is mixed in. This allows fresh air to continuously enter the spaces and reduces the amount of energy required to condition the supply air required completely from the outdoor air. 

The new concept, in this paper, suggests using the exhausted air to cool the lower surface of TEG modules, which are placed in series and heated at their top surface by the oil itself, which is heated by solar irradiation.

## 3. Parametric Analysis and Recommendations

A parametric analysis of TEG thermal behavior exposed to various conditions is performed. The aim is to generate a considerable temperature gradient in the TEG module. It is well known that generating power in TEGs requires the generation of a temperature gradient; nonetheless, one way to generate power is by preserving the temperatures at the two surfaces of the TEG. However, fixing the temperature at the two surfaces requires auxiliary outward resources that require power. Therefore, several loading and boundary conditions are considered as detailed below. Moreover, the relevant parametric study for each case will be considered under the steady-state condition along with assuming that the TEG is a plane wall whose conduction is steady and one-dimensional.

**Figure 4:**
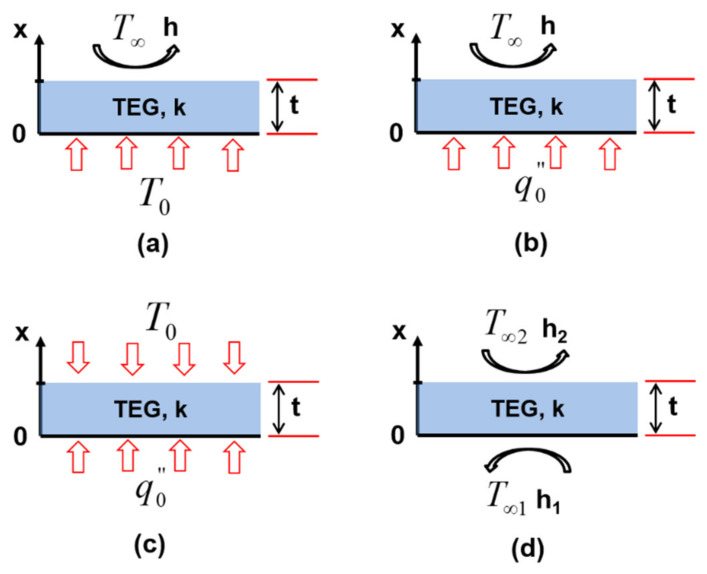
(**a**) Configuration 1; (**b**) configuration 2; (**c**) configuration 3; and (**d**) configuration 4.

Figure 4 depicts the different considered configurations schematically.

Firstly, in configuration one (Figure 4a), the TEG of thickness t and thermal conductivity k is exposed to a constant temperature $T_{0}$ at its hot side and simultaneously cooled by air with a convective coefficient h and temperature $\;T_{\infty }$. 

Secondly, in configuration two (Figure 4b), the TEG is exposed to a constant heat flux $q_{0}^{\prime \prime }$ at its hot side and simultaneously cooled by air with the convective coefficient h and temperature $\;T_{\infty }$. 

Thirdly, in configuration three (Figure 4c), the TEG is heated at its hot side with a constant heat flux $q_{0}^{\prime \prime }$ and simultaneously exposed to a constant temperature $T_{0}$ at its cold side. 

Finally, in configuration 4 (Figure 4d), the TEG is heated at one side with air with a temperature $T_{\infty 1}$ and convective heat transfer coefficient $h_{1}$ while being simultaneously cooled using air with a temperature $T_{\infty 2}$ and convective heat transfer coefficient $h_{2}$ at the other side.

Based on the aforementioned assumptions, the well-known heat diffusion equation is reduced to the following:

(1) $$\frac{d^{2}T}{dx^{2}}=0$$

The temperature distribution and the temperature gradient shown in the previous configurations can be attained by solving the differential equation of the heat diffusion equation (Equation (1)); however, they will differ according to the boundary conditions. In all previously mentioned configurations, integrating the reduced form of the heat diffusion equation with respect to x yields the linear variation (Equation (2)) where the two constants A and B are functions of the boundary conditions:

(2) $$T\left(x\right)=Ax+B$$

The boundary conditions, temperature distribution, and temperature difference corresponding to each configuration are meticulously detailed below:

**Configuration 1**

(3a) $$T\left(0,\theta \right)=T_{0}$$

(3b) $${\left.-k\frac{dT}{dx}\right|}_{x=L}=h\left[T\left(t,\theta \right)-T_{\infty }\right]$$

(3c) $$T\left(x\right)=\frac{h{\left(T_{\infty }-T\right)}_{0}}{K+ht}x+T_{0}$$

(3d) $$\Delta T=\frac{ht\left(T_{0}-T_{\infty }\right)}{k+ht}$$

**Configuration 2**

(4a) $${\left.-k\frac{dT}{dx}\right|}_{x=0}=q_{0}^{\prime \prime }$$

(4b) $${\left.-k\frac{dT}{dx}\right|}_{x=L}=h\left[T\left(t,\theta \right)-T_{\infty }\right]$$

(4c) $$T\left(x\right)=-\frac{q_{0}^{\prime \prime }}{k}x+T_{\infty }+\frac{ht+k}{hk}q_{0}^{\prime \prime }$$

(4d) $$\Delta T=\frac{q_{0}^{\prime \prime }t}{k}\;$$

**Configuration 3**

(5a) $${\left.-k\frac{dT}{dx}\right|}_{x=0}=q_{0}^{\prime \prime }$$

(5b) $$T\left(t,\theta \right)=T_{0}$$

(5c) $$T\left(x\right)=-\frac{q_{0}^{\prime \prime }}{k}x+T_{0}+\frac{q_{0}^{\prime \prime }}{k}t$$

(5d) $$\Delta T=\frac{q_{0}^{\prime \prime }t}{k}\;\;$$

**Configuration 4**

(6a) $${\left.-k\frac{dT}{dx}\right|}_{x=0}=h_{1}\left(T_{\infty 1}-T\left(0\right)\right)$$

(6b) $${\left.-k\frac{dT}{dx}\right|}_{x=L}=h_{2}\left(T\left(t\right)-T_{\infty 2}\right)$$

(6c) $$T\left(x\right)=\frac{h_{1}h_{2}\left(T_{\infty 2}-T_{\infty 1}\right)}{k\left(h_{1}+h_{2}\right)+h_{1}h_{2}t}x+T_{\infty 1}+\frac{kh_{2}\left(T_{\infty 2}-T_{\infty 1}\right)}{k\left(h_{1}+h_{2}\right)+h_{1}h_{2}t}$$

(6d) $$\Delta T=\frac{h_{1}h_{2}\left(T_{\infty 1}-T_{\infty 2}\right)}{k\left(h_{1}+h_{2}\right)+h_{1}h_{2}t}t\;\;$$

Based on the aforementioned resulting equations, a parametric analysis is performed so that the increase of temperature difference across the TEG plate can be identified and recommended. The observed recommendations are concisely reported in Table 1.

**Figure 5:**
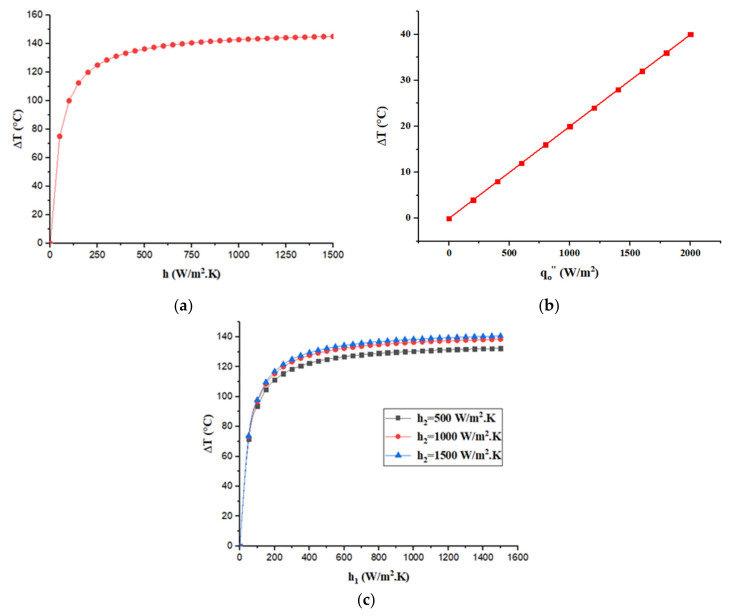
Variation of temperature difference as function of relevant parameters:(**a**) Configuration 1; (**b**) configuration 2 and 3; and (**c**) configuration 4.

Figure 5 presents the variation of temperature difference for the four configurations with respect to relevant parameters.

Figure 5a shows that for configuration 1 the temperature difference achieved 145 °C at 1500 W/m^2^ K convection heat transfer coefficient. While, Figure 5b presents the variation of temperature difference with respect to heat flux for both configurations 2 and 3. The maximum temperature difference obtained is 40 °C which is lower than configuration 1 and configuration 4 which is presented in Figure 5c. Regarding configuration 4 the maximum temperature difference achieved is 140 °C at h_1_ and h_2_ of 1500 W/m^2^ K. 

Both Configurations 1 and 4 have relatively similar temperature differences (at h_1_ and h_2_ = 1500 W/m^2^ K) from mathematical point of view. However, from practical point of view it is too hard to have a constant surface temperature as of configuration 1 which needs very high heat flux. This makes configuration 4 more preferable than configuration 1, 2 and 3.

Observations of the four investigated cases reveal that configuration four delivers the highest power. As detailed and revealed in Table 1, convection coefficients exist on both sides of the plate. Hence, it is crucial for an engineer or a designer to select the most appropriate convective heat transfer coefficients. It should be known that configuration four is widely encountered in engineering practice. Configurations two and three can also be encouraged to be used in applications containing extreme fluxes. Lastly, configuration one can also be enhanced where high temperatures exist in engineering applications.

## 4. Innovative Concept and Calculations

**Figure 6:**
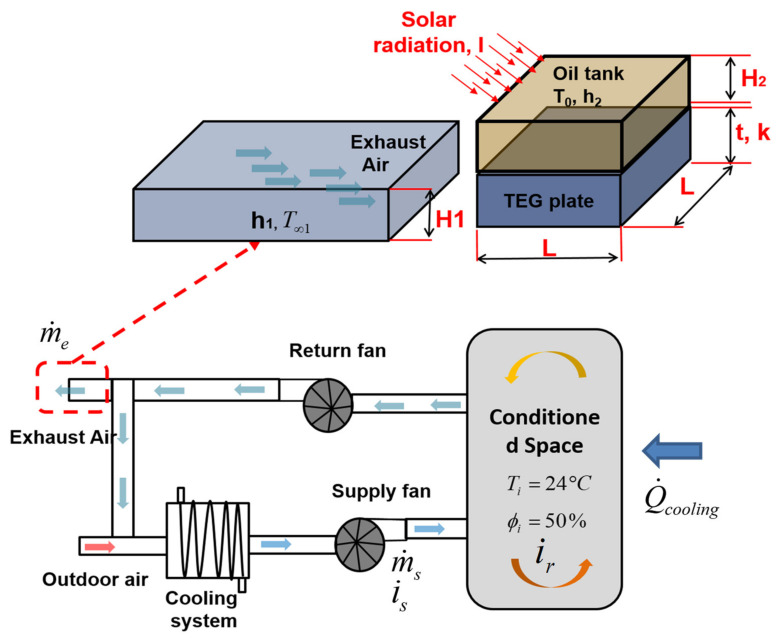
Schematic of the suggested concept of power generation.

As mentioned above, the parametric investigation uncovered that configuration four conveys the highest temperature gradient compared to the other configurations. Therefore, an innovative system is investigated by using solar radiation and the exhaust airflow (Figure 6). The proposed system is composed of an oil tank appended to its bottom flat plate where various TEG modules are placed in series; however, the upper transparent surface is visible to sun radiation. Moreover, the exhausted air of HVAC system is used to cool the bottom surface of the plate, contrarily, the upper TEG modules are heated by the hot oil which heated due to solar radiation. Therefore, a temperature gradient will be induced and will be converted to electrical power.

The following equation (Equation (7)) describes the mass flow rate of the exhausted air [30]:

(7) $${\dot{m}}_{e}=E{\dot{m}}_{s}$$

where, *E* is the relative fraction of exhausted air that depends on the specified conditioned space (housing, hospitals, etc.). The ${\dot{m}}_{s}$ represents the mass flow rate of the supplied air and calculated form the following equation [30]:

(8) $${\dot{m}}_{s}=\frac{{\dot{Q}}_{cooling}}{i_{r}-i_{s}}$$

where

- ${\dot{Q}}_{cooling}$: conditioned space cooling load.
- $i_{r}$: Specific enthalpy of air inside the room.
- $i_{r}$: Specific enthalpy of supplied air.

Afterward, the temperature and the relative humidity inside the conditioned space are estimated at 24 °C and 50% respectively as it is illustrated on ASHRAE (American Society of Heating, Refrigerating and Air Conditioning Engineers) standards [30] which leads to an enthalpy of 48 kJ/kg. Then, the enthalpy of the supplied air is estimated at 32 kJ/kg by considering a sensible heat factor of 0.8 and a supply temperature of 13 °C.

Then, the enthalpy of supplied air is estimated at 32 kJ/kg for a sensible heat factor of 0.8 and a supply air temperature of 13 °C. Considering the exhausted airflow below the TEG plate, the infinite velocity is calculated as follows [33]:

(9) $$\rho \cdot u_{\infty 1}=\frac{4{\dot{m}}_{e}}{\rho \pi D_{h}^{2}}$$

where ρ is the density of air and $D_{h}$ the hydraulic diameter calculated from the following relation [33]:

(10) $$D_{h}=\frac{4S}{P}=\frac{2\left(H_{1}L\right)}{H_{1}+L}$$

Later, the Reynolds number at the edge of the bottom plate is estimated as follows [33]:

(11) $${Re}_{L}=\frac{u_{\infty 1}L}{\mu }=\frac{4{\dot{m}}_{e}L}{\pi D_{h}^{2}\mu }$$

where μ is the dynamic viscosity of air. To estimate the heat transfer coefficient $h_{1}$ of air at the lower surface of the TEG plate, the flow regime should be checked: (i) The flow is considered as a Laminar for $Re_{L}$ lower than 5 × 10^5^ and (ii) the flow is mixed (Laminar then turbulent) when $Re_{L}$ is greater than 5 × 10^5^. Table 2 presents the correlation that can be used to estimate the Nusselt number and then the convective heat transfer coefficient [33]:

**Table 2:** Nusselt number and convection heat transfer coefficient correlations.

Flow Nature	Correlation
Laminar flow	Nu1=h1Lk=0.664ReL0.5Pr1/3
Mixed flow	Nu1=h1Lk=0.037ReL4/5−871Pr1/3

where $Nu_{1}$ represents the Nusselt number, k is the thermal conductivity, and Pr is the Prandtl number. 

The energy balance on the oil side is presented by the following equation [33]:

(12) $${\dot{E}}_{in}-{\dot{E}}_{out}+{\dot{E}}_{g}={\dot{E}}_{st}$$

where

- ${\dot{E}}_{in}$ represents the rate of energy “in” to the oil
- ${\dot{E}}_{out}$ The rate of energy out from oil
- ${\dot{E}}_{g}$ is the rate of energy that could be generated from the oil
- ${\dot{E}}_{g}$ is the rate of storage energy.

According to the present case, the rate of energy to the oil is equal to the transmitted solar radiation as presented in the equation below:

(13) $${\dot{E}}_{in}=\tau IL^{2}$$

where τ is the transmission coefficient of the transparent upper surface of the oil tank, I represents the solar radiation, and L is the side of the oil tank.

The rate of energy out from the system “${\dot{E}}_{out}$” is considered to be conducted from the system through the four walls of the tank with the ambient air. In addition, the rate of energy out is conducted by convection through the base of the tank composed of the TEG modules. Therefore, the rate of energy out is presented as follows:

(14) $${\dot{E}}_{out}=4H_{2}LU_{ins}\left(T_{o}-T_{a}\right)+L^{2}U_{b}\left(T_{o}-T_{\infty 1}\right)$$

where $H_{2}$ is the height of the oil tank, $U_{ins}$ is the overall heat transfer coefficient that represents the wall insulating of the oil tank, $T_{o}$ is the temperature of oil in the tank assume uniform, $T_{a}$ is the ambient temperature, $T_{\infty 1}$ is the temperature of the air that follows bellow the bottom plate, and $U_{b}$ is the overall heat transfer coefficient that represents the convection and conduction thermal resistance between the bottom plate and the oil and air and calculated by the following equation [33]:

(15) $$U_{b}=\frac{1}{\frac{1}{h_{2}}+\frac{t}{k}+\frac{1}{h_{1}}}$$

Then at a steady-state and no heat generation, it is as follows:

(16) $${\dot{E}}_{in}={\dot{E}}_{out}$$

And then

(17) $$T_{o}=\frac{IL+4H_{2}U_{ins}T_{a}+LU_{b}T_{\infty 1}}{4H_{2}U_{ins}+U_{b}L}$$

Then substituting the different parameters obtained above ($h_{1}$, $T_{\infty 1}$, $h_{2}$, $T_{o}$) in Equation (6d), the temperature difference across each TEG module is finally estimated by the following equation:

(18) $$\Delta T=\frac{h_{1}h_{2}\left(T_{o}-T_{\infty 1}\right)}{k\left(h_{1}+h_{2}\right)+h_{1}h_{2}t}t$$

Using the temperature gradient across the TEG, the electric power generated $P_{g}$ is estimated by the following equation [34]:

(19) $${\left(\frac{P}{\Delta T^{2}}\right)}_{ref}=\frac{P_{g}}{\Delta T^{2}}$$

where ${\left(\frac{P}{\Delta T^{2}}\right)}_{ref}\;$ is the reference ratio of power and temperature given by the manufacturer of the thermoelectric generator.

Table 3 represents the parameters that have been fixed in order to study the thermal behavior and the TEG power. 

**Figure 7:**
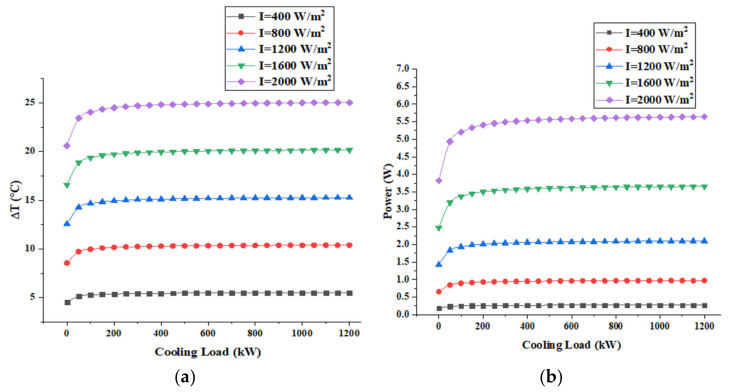
Variation of (**a**) temperature difference across each TEG module; (**b**) power generated by the assembly of TEG modules in the function of the cooling load.

Figure 7 shows the variation of the temperature difference across each TEG module in addition to the total power obtained by the suggested generator as a function of the space cooling load and different solar radiation.

As shown in Figure 7, the temperature difference across each TEG module increases slightly when the cooling load is increased and increases significantly with increasing solar radiation. As illustration for a solar radiation of 400 W/m^2^ when the cooling load varies from 20 to 500 kW, the temperature difference across each TEG module varies from 4.5 to 5.5 °C and the power generated by the assembly varies from 0.18 to 0.27 W (50% increase in power generated). For a solar radiation of 2000 W/m^2^ (for applications with solar concentration) when the cooling load varies from 20 to 500 kW, the temperature difference across each TEG module varies from 20.5 to 25 °C and the power generated by the assembly varies from 3.8 to 5.64 W (48% increase in power). 

As for the energy conversion efficiency of the TEGs, which is estimated by the ratio of the generated electric power over the thermal energy transferring through conduction on the TEG. The efficiency increases from about 0.5% when temperature difference across TEG is 4.5 ℃ to 2% when the temperature difference is 25 ℃.

Indeed, when the cooling load increases, the supplied mass flow rate and the exhausted air increase. Therefore, the convective heat transfer coefficient will also increase at the lower face of the bottom plate. As a result, it leads to an increase in the temperature difference across each TEG module, as shown in the parametric analysis presented above. However, the heat losses of the oil in the tank will increase and then decreases its temperature when the convective heat transfer coefficient at the lower surface of the bottom plate increases. For this reason, the TEG power of the assembly and the temperature difference will be directly affected and dampened.

## 5. Economic and Environmental Concerns

Economic and environmental benefits are recognized by estimating the money saved and amount reduced of CO_2_ gas. In order to perform the economic and environmental study, it is considered that 48 TEGs are attached in a square duct walls (16 TEG in 3 walls and the fourth wall is the bottom wall which can’t gain the solar radiation). 

However, the energy saved $E_{s}$ by the recovery system is estimated by the following equation:

(20) $$E_{s}=P_{g}\times N_{TEG}\times 30\times 24/1000$$

where $N_{TEG}$ is the number of TEGs attached and the constant is the conversion from W to kWh/month. 

**Figure 8:**
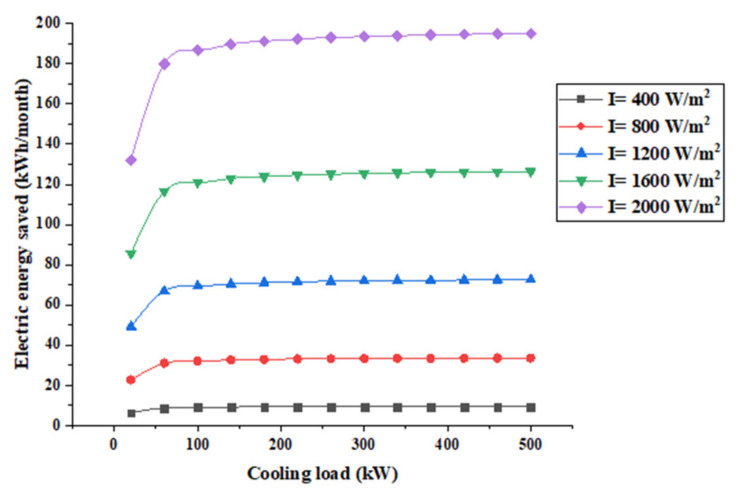
Electric energy saved by TEGs.

Figure 8 shows the electric energy saved per month. It shows that when solar radiation is 400 W/m^2^ and the cooling load varies from 20 to 500 kW, the energy saved varies from 6.4 kWh/month to 9.4 kWh/month. This increases with an increase in solar radiation to 2000 W/m^2^ and when the cooling load varies from 20 to 500 kW, the energy saved varies from 132 kWh/month to 195 kWh/month. This means that varying the solar radiation has a high impact of energy saved compared to a low impact of the cooling load.

The money saved MS by the system, which is equal to the cost of the energy saved, is estimated as follows:

(21) $$MS=E_{s}.C_{1kwh}$$

where $C_{1kwh}$ is the cost of one-kilowatt hour, which is considered based on its price in Lebanon, presented in Table 4.

**Figure 9:**
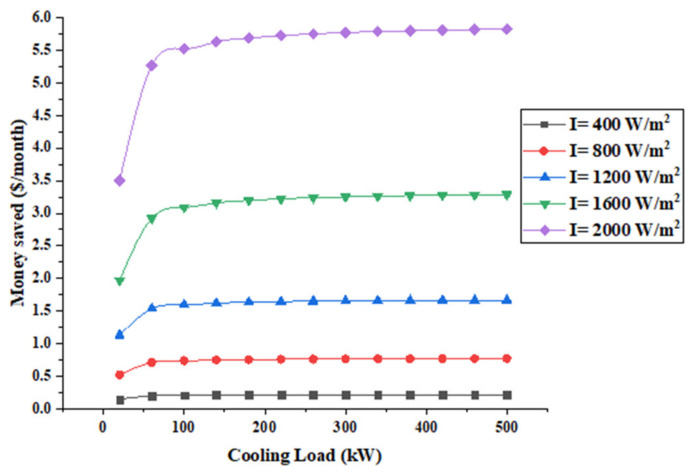
Money saved by the system.

Figure 9 presents the money saved as a function of the cooling load for different solar radiation.

It shows that the system is capable of approximately reducing the electric bill by $6/month at a 2000 W/m^2^ solar radiation, which decreases to about $1.7/month at 1200 W/m^2^. Furthermore, when the solar radiation is reduced by 40% the money saved is reduced by 70%. It should be noted that very little money is saved when the solar radiation is 400 W/m^2^, which is a result of the low power generated by thermoelectric generators. This implies that such system is more suitable for concentric systems. 

**Figure 10:**
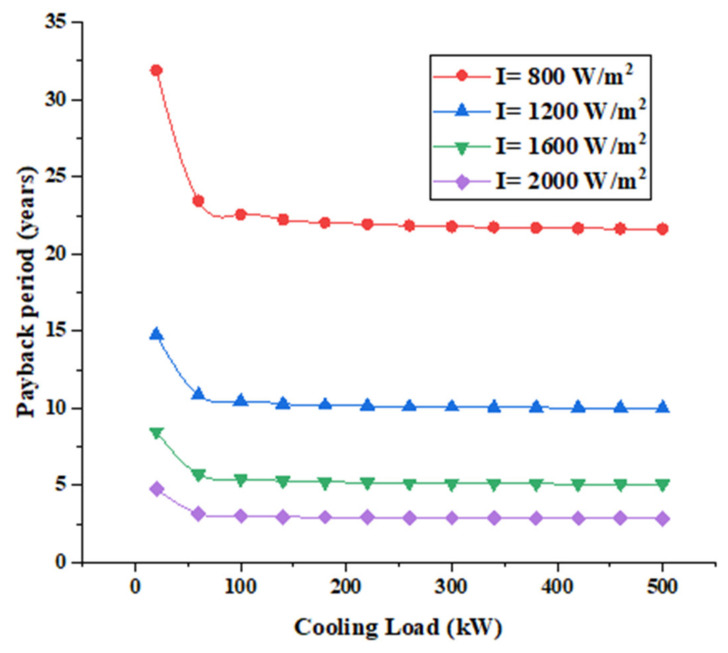
Payback period of the recovery system.

For the payback period calculations, the cost of the recovery system should be calculated. The recovery system is mainly composed of TEGs and a 40 cm × 40 cm plate that costs $200. It should be noted that 48 “SP 184827145 SA” TEGs are attached at three walls of a duct of 40 mm × 40 mm dimensions. Then the payback period PbP is defined as the fraction of the cost of the system over the money saved.

(22) $$PbP=\frac{C_{sys}}{MS}$$

where $C_{sys}$ is the total cost of the recovery system. Figure 10 below shows that payback period of the system under different conditions.

Figure 10 shows that when the solar radiation is 2000 W/m^2^, the payback period is about 3 years which increases to about 5 years when the solar radiation is 1600 W/m^2^. The reduction of solar radiation by 20% leads to a 66% increase in the payback period. The payback period of the system when the solar radiation is 400 W/m^2^ is very high (about 77 years) that’s why it is not presented in Figure 10 above. In addition, when the solar radiation is more than 1200 W/m^2^ the payback period is less than 15 years. The most acceptable payback period is for solar radiation more than 1600 W/m^2^ in which it is less than 5 years with cooling load greater than 100 kW.

Finally, regarding the environmental concerns, the reduced amount of CO_2_ gases $M_{CO2-reduced}$ by the system is estimated as follows.

(23) $$M_{CO2-reduced}=E_{s}.M_{CO2-released}$$

where $M_{CO2-released}$ is the amount of CO_2_ gas released to generate one-kilowatt hour, which is considered to be 0.47 kg/kWh in Lebanon [34]. 

**Figure 11:**
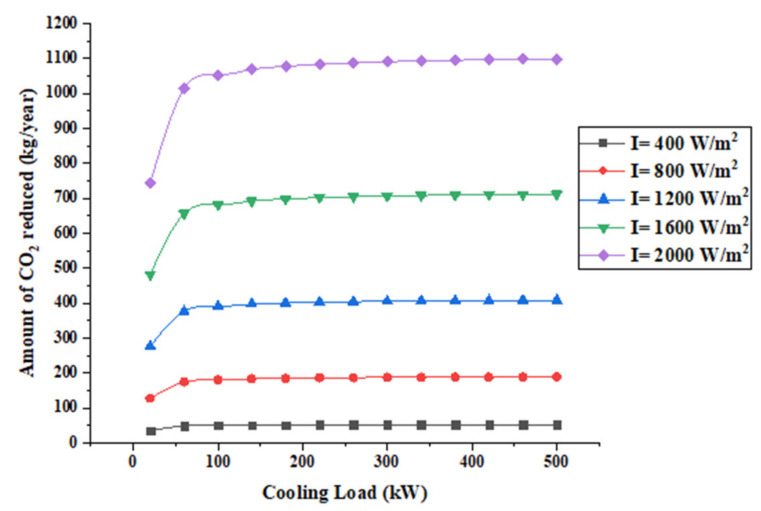
Amount of CO_2_ gases reduced per year.

Figure 11 shows the amount of CO_2_ gas reduced per year.

It also shows that the system can reduce about 1 ton yearly at a solar radiation of 2000 W/m^2^. Whereas, when the solar radiation is 1200 W/m^2^ the amount of CO_2_ gas reduced is about 0.5 ton/year. 

## 6. Conclusions

Energy recovery and renewable energy are two of the main fields of studies of researchers. Combining both technologies in a hybrid heat recovery system is a real challenge. In this manuscript, a new concept of power generation from TEGs using the exhaust airflow of all-air HVAC systems and sun radiation is presented. An appropriate thermal modeling of the system is performed as well. It is shown that for a solar radiation of 2000 W/m^2^ and a space cooling load of 20 kW, a 40 × 40 cm^2^ flat plate can generate 3.8 W of electrical power. In addition to that, the economic study shows that the system saves about $6 monthly with a 3-year payback period at 2000 W/m^2^ and environmentally it reduces about 1 ton of CO_2_ emissions yearly.

## Figures and Tables

**Table 1:** Summary of recommendations.

Configuration	Details	Recommendations	Parameters Range
**1**	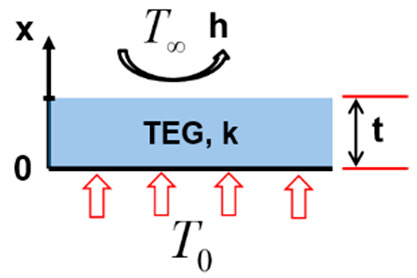	High convective heat transfer coefficient and high thickness “t” Low thermal conductivity and temperature at the cold side of the TEG module.	$T_{\infty }=20\;℃$ $T_{0}:100\to 150\;℃$ t:1 →10 cm $k:0.2\to 10\;\frac{W}{m.K}$
**2**	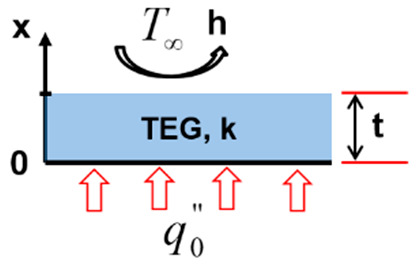	High thickness and heat flux at the hot surface of TEG. Low thermal conductivity of TEG.	$T_{\infty }=20\;℃$ t:1 →10 cm $k:0.2\to 10\;\frac{W}{m.K}$ $q_{0}^{’’}:0\to 2000\;\frac{W}{m^{2}}$
**3**	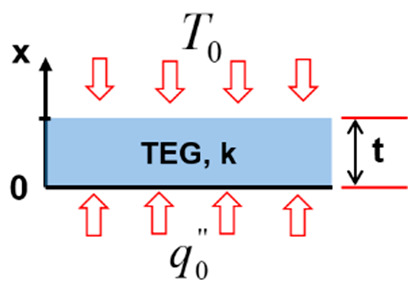	High thickness and high heat flux at the hot surface of TEG Low thermal conductivity of TEG module	$T_{\infty }=20\;℃$ t:1 →10 cm $k:0.2\to 10\;\frac{W}{m.K}$ $q_{0}^{’’}:0\to 2000\;\frac{W}{m^{2}}$
**4**	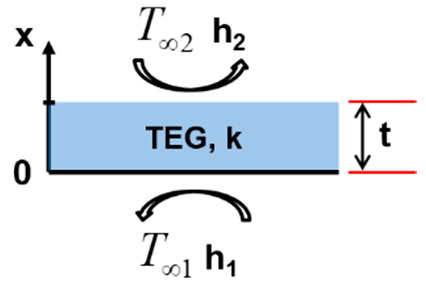	High thickness, high temperature of the hot fluid, high connective coefficient of the hot and cold side of TEG module Low thermal conductivity and low temperature of the cold fluid	$T_{\infty 2}=20\;℃$ $T_{\infty 1}:100\to 150\;℃$ $h_{1}:0\to 1500\;\frac{W}{m^{2}.K}$ t:1 →10 cm $k:0.2\to 10\;\frac{W}{m.K}$

**Table 3:** Fixed parameters of the system.

Variables	Value	Unit
Room temperature	24	°C
Fraction of exhausted air “E”	0.4	-
Height of the exhaust duct “H1”	0.1	m
The height of oil tank	0.1	m
The length of bottom plate composed of TEG	0.4	m
The width of bottom plate composed of TEG	0.4	m
Heat transfer coefficient $h_{2}$, [33]	200	W/m^2^ K
The overall heat transfer coefficient of insulation $U_{ins}$, [33]	8.4	W/m^2^ K
Ambient temperature $T_{a}$	30	°C
Thickness of TEG, [35,36]	0.12	m
Thermal conductivity of TEG, [35,36]	0.3	W/m·K

**Table 4:** Cost of one-kilowatt hour in Lebanon [34].

Electric Rates in One Month	Cost ($/kWh)
0–99 kWh/month	0.023
100–299 kWh/month	0.037
300–399 kWh/month	0.053
400–499 kWh/month	0.08
>500 kWh/month	0.133
